# Gender-Related Issues Among Women Raising Children: A Cross-Sectional Study Comparing Pre- and Post-pandemic Experiences

**DOI:** 10.7759/cureus.80912

**Published:** 2025-03-20

**Authors:** Sae Nakaoka, Hiromi Kawasaki, Satoko Yamasaki, Zhengai Cui, Mari Murakami, Sayo Mukaishima, Yuan Li

**Affiliations:** 1 Department of Health Science, Graduate School of Biomedical and Health Sciences, Hiroshima University, Hiroshima, JPN; 2 Department of Epidemiology and Public Health, School of Humanities and Management, Guangdong Medical University, Dongguan, CHN

**Keywords:** anger, childcare, fatigue, gender equality, pandemic, stress

## Abstract

Background

Despite being embraced worldwide, gender equality in childcare activities has not yet been achieved. Pandemics can negatively affect the health of women engaged in child-rearing. We surveyed women raising children both before and after the COVID-19 pandemic. The purpose of this study was to identify the effects of stress and fatigue levels in women through a comparison of surveys conducted before and after the pandemic and to determine the childcare requirements necessary for women to play an active role in society and achieve gender equality.

Methods

The surveys were conducted in 2017 and 2021. The target population consisted of 150 women with children aged 0-3 years. The survey items included basic attributes, situations in which they felt angry while raising their children, and degree of fatigue. To compare the data before and after the pandemic, a chi-square test, no correspondence t-test, and Wilcoxon's rank sum test were conducted on the survey items for the 2017 and 2021 groups.

Results

The average age of the mothers before and after the pandemic was 33 years, and most were from nuclear families. They relied on childcare support for more than an hour's drive. The number of women who worked or took childcare leave increased significantly after the pandemic (p=0.025). There were no significant differences in stress or fatigue levels before and after the pandemic. A lower number of women became angry when their children cluttered up the house (p=0.031) or were difficult when it was time to go home (p=0.008) after the pandemic.

Conclusions

Women’s stress and fatigue did not differ during the pandemic, and fewer women became angry about their children after the pandemic. Women raising children had lack of sleep and fatigue accumulation regardless of the pandemic. It is important for couples to discuss maternal fatigue on an ongoing basis, beginning when planning pregnancy. Rapid resolution of the challenges women face in raising children will achieve gender equality.

## Introduction

Gender ideologies differ according to state policies regarding work and family [1]. In the 1990s, the number of nuclear families in Japan increased and Japanese women began to advance in society because of the inclusion of gender equality in the Employment Act. Despite changes in family structure, women are still the primary providers of childcare and experience considerable childcare-related stress. Japan has witnessed a major decline in birth rate and population. As a result, the Japanese government implemented childcare support measures such as “angel plans.” To prevent population decline, the Japanese government considered promoting women in the workplace to maintain the Japanese workforce. This initiative caused numerous children to spend time in nursery schools. Japanese women's workforce is not very different from that of other countries, but their rate of occupying managerial posts is lower than that in other countries [2]. Japanese women cannot step up in their careers.

In 2015, the United Nations announced its Sustainable Development Goals, 17 of which should be achieved by 2030. These goals include gender equality and empowerment of all women and girls [3]. More than 60% of the Japanese population rejects the notion that men should work and women should stay at home [4]. Therefore, the status of gender equality is changing.

However, at odds with gender equality, women are still largely responsible for housework and childcare. In a survey involving Japanese men and women, 80% of respondents opined that men enjoy more privileges [2], and more women than men held this opinion [2]. Although the number of Japanese men doing housework and childcare has increased, it is still lower than in Europe and the United States [5]. Couples with preschool children spend more time on childcare and housework, but women spend more than twice as much time on these activities as men [4]. Women are responsible for housework and childcare, and their responsibilities do not decrease as their children grow up [4].

Women with young children experience cumulative stress related to childcare because raising their children is a new experience. The isolation of women raising children is a major problem in Japan [6], and women do not have sufficient opportunities to relieve their stress or share concerns associated with childcare.

The COVID-19 pandemic began in December 2019 and negatively affected the physical and social health of women, because of factors such as limited work, changes in response to pregnancy and childbirth, and increased domestic violence [7]. The pandemic was also a period when worse mental health status was observed among women. Because of pandemic restrictions, women were unable to give birth accompanied by their husbands or to receive visitors. In addition, after childbirth, women could not spend enough time in the hospital and did not have the opportunity to receive childcare support from healthcare providers. Consequently, the number of new mothers with depression and anxiety symptoms increased during the pandemic [8].

Previous studies that have evaluated the effects of the pandemic on women found that the pandemic increased their stress levels [9]. The government recommended telecommuting and a reduction in working hours to control the pandemic. Consequently, women worked from home and cared for their children, which led to increased stress and fatigue. However, women’s isolation has always been an issue even before the pandemic. Most studies assessing the effect of the spread of infectious diseases in women were conducted during the pandemic. Therefore, whether women experienced stress and fatigue even before the pandemic or whether it occurred as a result of the pandemic remains unknown.

The purpose of this study was to identify the effects on stress and fatigue levels of women through the results of surveys conducted before and after the pandemic, and to determine from a childcare perspective what is required for women to play an active role in society and to achieve gender equality.

This article was presented as a meeting abstract at the 27th East Asian Forum of Nursing Scholars on March 7, 2024.

This article was posted on the Research Square Preprint Server on December 17, 2023.

## Materials and methods

Design

This was an exploratory cross-sectional study. The results of surveys conducted in 2017 and 2021 using the same questionnaire were used as quantitative and secondary data. Secondary quantitative data were used to determine the impact of the pandemic by comparing situation-induced anger and childcare exhaustion among mothers who used the nonprofit Child Care Support Facility A (referred to as the square) in 2017 (before the pandemic) and 2021 (after the pandemic).

Sample

The study participants were all square participants. Since the square was a facility where mothers raised children voluntarily, the participants were not fixed. The survey forms were distributed to mothers who agreed to take the survey when they visited the square; 150 copies were distributed for each study period in 2017 and 2021. The exclusion criteria were defined as missing responses.

Data collection and analysis

From May 1 to July 31, 2017, and October 1 to November 30, 2021, an anonymous, self-administered questionnaire survey was conducted with 150 participants at the square for project reporting and budget planning purposes. The Japanese government has encouraged the establishment of child-rearing support facilities as part of its maternal and childcare programs. Mothers can use the square to care for their children aged 0-3 years. The responses obtained were used secondarily, with the permission of a representative of the nonprofit organization.

The 2021 survey was completed by 116 (77.3%) of the 150 respondents. Ninety-seven participants (64.7%) provided complete responses and were included in the analysis. The 2017 survey was completed by 103 participants (68.7%) of the 150 respondents. Ninety-three participants (62.0%) answered completely and were included in the analysis. G*Power was used to confirm that the sample size was sufficient for a valid analysis.

Contents of the survey

The survey items included basic attributes (mother’s age, family structure, childcare support, working status, frequency of using the square, sleep duration, wake-up time, and presence or absence of childcare problems), anger triggered by situations, childcare fatigue, and sense of urgency.

Situations triggering anger were configured for 10 daily childcare situations to make it easier for the subject to answer the questions. NPO staff, who typically provide childcare counseling at childcare support facilities, were interviewed, and the results were reflected in the questionnaire items: 1. When the child does not get up easily in the morning; 2. When the child has difficulty sleeping at night; 3. When the child wakes up in the middle of the night; 4. When the child takes a long time to finish a meal; 5. When the child eats only what he/she likes and leaves behind what he/she dislikes; 6. When the child is resistant to changing clothes; 7. When the child has difficulty bathing; 8. When the child clutters the house; 9. When the child is resistant to going home, even when it is time to leave; and 10. When the child cries for extended periods. The participants responded to each of these situations with "I get angry", "I get angry sometimes", or "I do not get angry".

For childcare fatigue, a questionnaire called Jikakusho-shirabe, which assesses feelings of drowsiness, instability, uneasiness, localized or dull pain, and eyestrain, was used, as previously described [10]. This questionnaire was confirmed to be valid and reliable by the Japan Society for Occupational Health.

The sense of urgency was calculated using excerpts from the Japanese version of the Type A Questionnaire for Japanese adults. From three scales, "aggression and hostility," "vigorous activity and urgency," and "speed and intensity of action", we used the 16 items from the "vigorous activity and time urgency" scale. A previously published questionnaire was used to assess the participants' sense of time urgency [11].

Data analysis

We conducted a cross-sectional survey. The 2021 and 2017 surveys included the same set of questions. For the questions about situations that make them angry, we combined “I get angry sometimes” with “I get angry” and used a two-tailed method with “I don’t get angry” and “I get angry sometimes.” A chi-squared test was conducted for the two test questions. Mothers’ age, sleeping duration, childcare fatigue, and sense of urgency were tested for normality using the Shapiro-Wilk test, and items with normality were subjected to an uncorrelated t-test. Statistical analyses were performed using SPSS version 25.0 (IBM Corp., Armonk, NY). The significance level for all tests was set at <5%.

Ethical approval and informed consent

This study was approved by the Ethics Committee of Hiroshima University (approval no. E2017-0962-03) and was conducted in accordance with the principles of the Declaration of Helsinki. The participants were provided with verbal and written explanations in advance. Submission of the completed questionnaire at the designated location was regarded as consent to participate in the survey and publish the results. Informed consent was obtained from all participants by the head of the nonprofit organization. Permission to use the data was obtained from the head of the nonprofit organization.

## Results

Characteristics of the respondents

Table 1 presents the main characteristics of the participants. Significant differences were observed in the working status between the two groups, referred to as “before the pandemic,” in 2017 and “after the pandemic” in 2021. Before and after the pandemic, the average age of the mothers was 33.3±4.49 and 33.0±5.17 years, respectively. Significantly more participants were either working or taking childcare leave after the pandemic (p=0.025). Regarding family members living together, 97.8% and 94.8% of the participants lived in nuclear families before and after the pandemic, respectively. In contrast, the presence of extended family members was reported by 2.20% and 5.20% of the respondents, respectively.

**Table 1:** Characteristics of mothers (N=93,97) Note: # indicates the chi-squared test. & indicates t-test without correspondence or Wilcoxon rank-sum test.

Characteristics	2017	2021	P-value	
Mother's age	33.3±4.49	33.0±5.17	0.638	&
Family structure				
Nuclear family	91 (97.8%)	92 (94.8%)	0.272	#
Extended family	2 (2.2%)	5 (5.2%)		
Distance of people you can rely on for childcare				
Less than 1 hour by car	23 (24.7%)	29 (29.9%)	0.425	#
At least 1 hour by car	70 (75.3%)	68 (70.1%)		
Do you have childcare support?				
Yes	91 (97.8%)	94 (96.9%)	0.520	&
No	2 (2.2%)	3 (3.1%)		
Working status				
Working or on childcare leave	19 (20.4%)	34 (35.1%)	0.025	#
Not working	74 (79.6%)	63 (64.9%)		
Frequency of using the square				
Less than four times a month	49 (52.7%)	62 (63.9%)	0.116	#
At least four times a month	44 (47.3%)	35 (36.1%)		
Sleep duration	6.4±1.10	6.3±1.00	0.368	&
Wake-up time				
The same time	69 (74.2%)	66 (68.0%)	0.350	#
Irregular times	24 (25.8%)	31 (32.0%)		
Presence or absence of childcare problems				
Yes	63 (67.7%)	74 (76.3%)	0.189	#
No	30 (32.3%)	23 (23.7%)		
Sense of time urgency	12.5±5.91	12.3±6.23	0.805	&

More than 90% of participants lived in nuclear families. Before and after the pandemic, 24.7% and 29.9% of mothers drove for less than an hour to someone they relied on for childcare and other needs, respectively. The proportions of mothers who drove for at least one hour by car were 75.3% and 70.1%, respectively. Approximately 70% of the mothers relied on someone within an hour’s drive or more for childcare. Before and after the pandemic, 52.7% and 53.9% of mothers used the square fewer than four times per month, respectively. Before and after the pandemic, the sense of urgency scores were 12.5±5.91 and 12.3±6.23.

Comparison before and after the pandemic

Table 2 summarizes the situations in which mothers experienced anger during parenting. When children cluttered the house before and after the pandemic, 49.5% and 34.0% of the mothers became angry, respectively. When their children refused to go home easily, 44.1% and 25.8% of the mothers became angry. There were significant differences before and after the pandemic when children cluttered up the house and refused to go home easily (p=0.031 and p=0.008, respectively).

**Table 2:** Anger triggered by situations (N=93,97)

Situations	2017	2021	P-value
When the child does not get up easily in the morning			
I don't get angry	86 (92.5%)	93 (95.9%)	0.315
I get angry sometimes	7 (7.5%)	4 (4.1%)	
When the child has a hard time sleeping at night			
I don’t get angry	48 (51.6%)	59 (60.8%)	0.201
I get angry sometimes	45 (48.4%)	38 (39.2%)	
When the child wakes up in the middle of the night			
I don’t get angry	84 (90.3%)	87 (89.7%)	0.885
I get angry sometimes	9 (9.7%)	10 (10.3%)	
When the child takes a long time to finish a meal			
I don’t get angry	33 (35.5%)	42 (43.3%)	0.271
I get angry sometimes	60 (64.5%)	55 (56.7%)	
When the child eats only what he/she likes and leaves behind what he/she dislikes			
I don’t get angry	47 (50.5%)	58 (59.8%)	0.200
I get angry sometimes	46 (49.5%)	39 (40.2%)	
When the child does not easily change clothes			
I don’t get angry	48 (51.6%)	61 (62.9%)	0.116
I get angry sometimes	45 (48.4%)	36 (37.1%)	
When the child has a difficult time bathing			
I don’t get angry	61 (65.6%)	73 (75.3%)	0.144
I get angry sometimes	32 (34.4%)	24 (24.7%)	
When the child clutters up the house			
I don’t get angry	47 (50.5%)	64 (66.0%)	0.031
I get angry sometimes	46 (49.5%)	33 (34.0%)	
When the child does not go home easily, even when it is time to go home			
I don’t get angry	52 (55.9%)	72 (74.2%)	0.008
I get angry sometimes	41 (44.1%)	25 (25.8%)	
When the child cries for extended periods of time			
I don’t get angry	66 (71.0%)	76 (78.4%)	0.242
I get angry sometimes	27 (29.0%)	21 (21.6%)	

The number of mothers who exhibited anger decreased after the onset of the pandemic. Furthermore, 7.5% and 4.1% of the mothers before and after the pandemic, respectively, were angry when their children did not get up easily in the morning. When their children had difficulty sleeping at night, 48.4% and 39.2% of the mothers experienced anger before and after the pandemic, respectively. Furthermore, 9.7% and 10.3% of mothers became angry when their children woke up in the middle of the night; 64.5% and 56.7% of mothers became angry when their children took a long time to finish a meal; and 49.5% and 40.2% of mothers became angry when their children ate only what they liked and left behind what they disliked before and after the pandemic, respectively. Moreover, 48.4% and 37.1% of mothers became angry when their children could not easily change their clothes, 34.4% and 24.7% of mothers became angry when their children had a hard time taking a bath, and 29.0% and 21.6% of mothers became angry when their children cried for extended periods before and after the pandemic, respectively.

Table 3 shows the mean scores for the subjective feelings of childcare-related fatigue before and after the pandemic. All mean fatigue scores decreased after the pandemic. No significant differences were observed between the groups. The scores before and after the pandemic were 13.4±5.13 and 13.3±4.60 for drowsiness, 10.5±4.60 and 9.8±4.35 for instability, 9.3±4.23 and 8.8±3.48 for uneasiness, 11.6±4.14 and 11.1±4.46 for localized or dull pain, and 9.0±4.21 and 8.6±3.69 for eyestrain.

**Table 3:** Mean scores of subjective feelings of childcare fatigue

Fatigue	2017	2021	p-value
Drowsiness	13.4±5.13	13.3±4.60	0.906
Instability	10.5±4.60	9.8±4.35	0.364
Uneasiness	9.3±4.23	8.8±3.48	0.642
Localized or dull pain	11.6±4.14	11.1±4.46	0.252
Eyestrain	9.0±4.21	8.6±3.69	0.716

## Discussion

We examined the main features of the mothers who participated in the surveys and compared the results before and after the pandemic to examine their daily child-rearing situations and fatigue. There were no significant differences in fatigue. Women raising children continue to face fatigue-related challenges, regardless of the pandemic. The pandemic has brought new challenges to women with respect to their daily parenting situations, suggesting that they face challenges in carving out time for themselves because of gender-based differences in parenting methods and responsibilities.

The following is a summary of the backgrounds of the participants in the two surveys. Notably, significant differences were observed in the number of working women who used the square. No significant differences were observed in the backgrounds or lives of the other participants.

Background of the subjects

The average age of the women in this study was close to the average age at childbirth in Japan, reflecting the current situation [12]. More than 90% of the participants had nuclear families, and approximately 5% belonged to three-generation families. In Japan, children are raised in nuclear families in 82.5% of cases and three-generation families in 13.3% of cases [13]. This study examined the current general situation in Japan. The distance covered by mothers to reach childcare support providers did not differ significantly before and after the pandemic. For >70%, the mothers’ childcare support was more than an hour’s drive away from home. During the pandemic, restrictions on movement as part of the infection control measures made it even more challenging for mothers to access childcare support systems. Furthermore, the square was closed and the number of users was restricted to curb the pandemic. Since human connections help alleviate worries [14], the pandemic exacerbated worry for women raising children; 80% of the women who participated in the surveys experienced childcare-related problems. They may not have received adequate childcare support from childcare providers or from the square before the pandemic. The average sleep duration was 6.4 hours before and 6.3 hours during the pandemic. Sleep duration and wake-up time did not differ significantly before and during the pandemic. The sleeping time for Japanese women was 7.35 hours in 2016 and 7.49 hours in 2021 [15]; therefore, the study participants had an hour less sleep than the average Japanese women. Overall, women raising children slept less, regardless of the pandemic. Regarding waking time, more than 70% of the participants woke up at a fixed time before the pandemic, but this number decreased after the pandemic. Delays in falling asleep and waking up were reported during the pandemic [16], which may have caused a shift in the chronobiological rhythms of women. This may have been exacerbated by the remote working setup and restrictions on going out and staying indoors during the pandemic, which may have affected their daily rhythms. An analysis of the number of women who made use of the square facility indicated a significant increase in the number of working women or those taking maternity leave during the pandemic compared to before the pandemic. During infectious disease outbreaks, we need to consider the effect of working from home and reduced working hours as part of infectious disease control measures. Immediately after the pandemic, women who were not raising children returned to their normal work. However, a high percentage of women raising children remain absent from work [17]. It is possible that the number of women who did not make use of the square because of work before the pandemic could now use that space, because of working from home and reduced working hours. Infection prevention measures provided women who could not previously use the square on weekdays with opportunities to use it.

Women’s anger and fatigue of childcare

This study focused on women’s anger as an emotional condition and fatigue levels. We examined the women’s health issues by assessing their daily anger and fatigue levels. The reasons for focusing on anger are discussed below: Mothers have a responsibility to care for their children, so they commonly become angry when they are unable to regulate their children’s behavior. Difficulty controlling anger negatively affects women’s health. Women undergo physical and emotional changes during pregnancy and childbirth. After delivery, a woman must take care of the child in addition to her regular responsibilities. Despite the physical and mental changes associated with pregnancy and childbirth, women are forced to engage in housework and childcare without adequate recovery time. Consequently, women are at risk of cumulative fatigue, which can lead to additional parenting stress [18].

Here, we discuss the anger of women raising their children in detail. The relationship between the ease of becoming angry and a sense of urgency has been demonstrated [19]. The average sense of urgency score for women in this study clearly indicates that these women were not particularly prone to anger. These scores were not significantly different before and during the pandemic; they were approximately 12 points, indicating that women did not have a high risk for a sense of time urgency compared with those in previous studies [20]. The anger levels when a child cluttered up the house and did not easily cooperate when it was time to go home showed significant differences. Compared with the pre-pandemic period, fewer women became angry about childcare during the pandemic. Other parenting situations showed no significant differences in how angry the women felt before and after the pandemic. We speculate that mothers did not get angry in these two situations as they no longer had to take their children to and from daycare, nor did they go to work because of work-from-home setup, restrictions on general movement, or voluntary refraining from going out as part of infection control measures, which gave them more time to spare. While women avoided going out, they had extra time for housework and childcare, particularly cleaning their houses [21]. The pandemic notably reduced the number of times women and their children went out. Use of the square in this study was restricted; other studies reported that shopping complexes were also closed. During the pandemic, women had no choice but to compromise with their children and did not become angry about childcare because they spent a long time with their children. However, excessive emotional control can also lead to high aggression [22]. In this study, not becoming angry while taking care of children meant not feeling angry with their children but trying to restrain anger; as a result, women experienced more stress. Therefore, further studies are required to examine the relationship between anger and stress.

Next, we examined women’s fatigue levels before and during the pandemic. The average scores for five groups of “Jikaku-sho shirabe” (group 1: drowsiness, group 2: instability, group 3: uneasiness, group 4: localized or dull pain; and group 5: eyestrain) did not differ significantly before and after the pandemic. Compared to the fatigue scores of postpartum women with hospital support, scores of instability and anxiety were relatively high, with no significant differences among the other groups [23]. Women were considered to have taken on more responsibility for protecting their children, as they took care of their own children. Even before the pandemic, fatigue was high among women raising children, suggesting that women constantly experienced fatigue.

The mean total scores for each of the five groups showed the following pattern: drowsiness > localized or dull pain > instability > uneasiness > eyestrain. The children in this study were young and unable to care for themselves. Women raising children must constantly take care of them and cut into their own sleeping hours to complete the remaining household chores and other activities. Drowsiness and localized or dull pain are believed to result directly from fatigue. Other symptoms include pain in specific areas of the body. These symptoms are associated with aging [24]. The average age of the participants indicated that other symptoms did not score higher than feelings of drowsiness and localized or dull pain.

The drowsiness subscale (group 1) asked about the presence or absence of physical and mental symptoms experienced by women when they were sleepy. The total score was higher because the participants slept less than the average woman before and after the pandemic [15]. Several studies have reported sleep challenges among women raising children. Sleep quality and sleep problems are associated with maternal anger [25]. Ongoing support is needed to overcome sleep deficits to decrease anger, which has not been emphasized because of the pandemic.

The instability subscale (group 2) asked women about the presence or absence of mental symptoms. We predicted that the total instability score would increase when women did not receive sufficient childcare support. Previous studies have shown that instability is related to social distancing from friends, family, and public facilities [6]. We hypothesized that women's instability would increase because they would have to limit their interactions with others during the pandemic. We did not refer to previous studies because the total instability scores were not significant before or after the pandemic. Before the pandemic, participants regularly attended the square and received childcare support. However, after the pandemic, they did not receive childcare support earlier because of infection control measures, putting them at risk of increased childcare-related problems and fatigue.

The uneasiness subscale (group 3) asked women about the presence or absence of headaches. In a study investigating the relationship between headaches and the pandemic, patients with migraines reported improved symptoms because they had more opportunities to review their lives by refraining from going out [26]. In this study, women’s instability did not decrease significantly; therefore, headaches were unaffected by the pandemic.

The localized or dull pain subscale (group 4) consisted of numerous physical symptoms. Working mothers must also care for their children, which can cause physical fatigue. During the pandemic, women were required to care for their children and work at home throughout the day. A previous study showed that women spent approximately three hours on housework, two hours on child-rearing, and 30 minutes on themselves before the pandemic [27]. However, they felt that the amount of housework increased and that the division of household chores was not equal after the pandemic [21].

The eyestrain subscale (group 5) encompassed numerous visual symptoms. During the pandemic, many people experienced eyestrain due to the use of electronic equipment for extended periods [28]; however, their total eyestrain scores before and after the pandemic did not differ significantly. In a previous study, subjects were surveyed regarding their digital device time, viewing distance, and purpose. Because a previous study reported that men and women with an average age of 35 years reported increased screen time during the pandemic, it was possible that the subjects in this study also had increased screen time. Our study disproved the hypothesis of increased eyestrain due to the use of equipment for extended periods during the pandemic.

In this study, we compared mothers’ parenting environments before and after the pandemic and found that women raising children experienced sleep deprivation and accumulated fatigue before the pandemic. Fatigue among women raising children was also emphasized during the pandemic; however, these problems did not arise specifically. Women raising children may be unaware of these issues. They and their partners need to talk to each other about their fatigue, starting at the time of planning the pregnancy and continuing it. They should share their sleep schedule with their partners. It is important for the partner to notice the accumulation of fatigue by observing their facial expressions and behavior, and to involve the partner in parenting and suggest sources of support. Nursing professionals should instruct both their partners about maternal fatigue as much as possible when they receive their Maternal and Child Health Handbook, when they visit the Obstetrics and Gynecology clinic, or when they come to childcare school or health checkups.

The government's policy on remote working and restrictions on free movement during the pandemic has revealed the following issues related to Sustainable Development Goal 5: Gender equality. These issues are also relevant to normal times.

During the pandemic, the government encouraged people to work from home, and both men and women adopted remote work. The advantage was that women who were initially unable to use the square because of employment could now use it according to their schedules. Another benefit was that both men and women could participate in raising children. However, many men only engaged in housework and childcare to the extent that it did not affect their work [4]. This highlights the differences in child-rearing methods and responsibilities between men and women. Restrictions were placed on the movement of childcare supporters, and the squares were closed. Women lost the childcare support they had previously received and had difficulty relieving their stress. Women previously made time for themselves by changing locations and schedules, but the curfew made it difficult for them to switch roles. Daily stress forced women raising children to suppress their anger and continue to suffer chronic fatigue.

Although the study findings suggested that women raising children had difficulty taking time for themselves at all times, this difficulty negatively affected their anger and fatigue levels. The existing literature emphasizes this as an effect of the pandemic [7-9] but our study did not suggest this. Our study highlights specific points that need to be improved immediately regarding gender equality in parenting households. The responsibilities and burdens of women raising children must be spread to achieve gender equality in childcare.

Strengths and limitations

The study analyzed in detail the parenting stress and fatigue of mothers, highlighted by the pandemic, and found that parenting stress did not occur as a result of the pandemic but was felt by mothers all the time. This suggests that people may still believe that the responsibility for child rearing lies with women, despite the desire to promote gender equality.

The number of respondents was limited because the study used a secondary survey for the operational planning of one childcare support facility. We need to increase survey participants and add cooperative facilities but the data on situations after the pandemic is valuable. We believe that the demographics of the users of the facility where the survey for the analysis was conducted reflect Japanese mothers and that generalizations can be made. Although the percentage of cooperation with the distributed survey forms was high, the impact cannot be ruled out because approximately 10% of incomplete responses were deleted. Reasons for missing responses included the use of scales. The fatigue and time urgency scales were used in the questionnaire. The scale cannot be used unless the number of questions cannot be adjusted and complete responses are not available. Mothers may have experienced difficulties answering multiple scales. Since men were not included in the study, the situations of women could not be compared to those of men, and this should be considered in the future. In this study, the limited number of child-rearing situations and the degree of anger were classified into three categories. In the future, further classification of child-rearing situations will be considered to establish a correlation between child-rearing situations and anger, as well as to make comparisons between different situations.

## Conclusions

By comparing the results of a survey of women raising children before and after the pandemic, we examined parenting-related stress and fatigue levels among women. Women raising children had high levels of stress and fatigue regardless of the pandemic. They have always experienced problems of lack of sleep and cumulative fatigue. It is important for couples to discuss maternal fatigue on an ongoing basis, beginning when planning pregnancy. To achieve gender equality in childcare, the responsibilities and burdens of women raising children must be shared more equally.
